# Implicit domain adaptation with conditional generative adversarial networks for depth prediction in endoscopy

**DOI:** 10.1007/s11548-019-01962-w

**Published:** 2019-04-15

**Authors:** Anita Rau, P. J. Eddie Edwards, Omer F. Ahmad, Paul Riordan, Mirek Janatka, Laurence B. Lovat, Danail Stoyanov

**Affiliations:** 1 Wellcome/EPSRC Centre for Interventional and Surgical Sciences (WEISS), University College London, London, UK; 2 Digital Surgery Ltd., London, UK

**Keywords:** Depth estimation, 3D reconstruction, Conditional GANs, Colonoscopy

## Abstract

**Purpose:**

Colorectal cancer is the third most common cancer worldwide, and early therapeutic treatment of precancerous tissue during colonoscopy is crucial for better prognosis and can be curative. Navigation within the colon and comprehensive inspection of the endoluminal tissue are key to successful colonoscopy but can vary with the skill and experience of the endoscopist. Computer-assisted interventions in colonoscopy can provide better support tools for mapping the colon to ensure complete examination and for automatically detecting abnormal tissue regions.

**Methods:**

We train the conditional generative adversarial network pix2pix, to transform monocular endoscopic images to depth, which can be a building block in a navigational pipeline or be used to measure the size of polyps during colonoscopy. To overcome the lack of labelled training data in endoscopy, we propose to use simulation environments and to additionally train the generator and discriminator of the model on unlabelled real video frames in order to adapt to real colonoscopy environments.

**Results:**

We report promising results on synthetic, phantom and real datasets and show that generative models outperform discriminative models when predicting depth from colonoscopy images, in terms of both accuracy and robustness towards changes in domains.

**Conclusions:**

Training the discriminator and generator of the model on real images, we show that our model performs implicit domain adaptation, which is a key step towards bridging the gap between synthetic and real data. Importantly, we demonstrate the feasibility of training a single model to predict depth from both synthetic and real images without the need for explicit, unsupervised transformer networks mapping between the domains of synthetic and real data.

## Introduction

Almost a fifth of the population will develop colorectal adenomas in their lifetime. However, the development from benign polyps into malignant cells is a process spanning several years [1]. Frequent screening using colonoscopy can prevent the development of colorectal cancers, but the procedure greatly depends on the proficiency of the operator and miss rates of precancerous lesions can be as high as 90% for non-expert endoscopists [2]. The quality of procedures is typically assessed through the adenoma detection rate of the endoscopist and the withdrawal time, but both measures have limitations. The former attests only a particular colonoscopist’s rate of polyp detection vis-à-vis other colonoscopists and thus provides minimal data about any individual procedure. The latter merely measures the amount of time performing the procedure. There is no enforcement of proportionate time allocation across regions.

To improve polyp detection rates and to standardize colonoscopy around the world, researchers have focused on two problems: (1) how to better detect polyps on observed endoluminal surfaces, and (2) how to determine whether the entire colon wall has been endoscopically observed. One approach to the first problem is to develop automatic polyp detection algorithms that evaluate images during colonoscopy and direct endoscopists’ attention to areas with a high probability of being polyps. The joint efforts of clinicians and scientists to develop public datasets, annotating thousands of images [3], gave scientists a foundation for developing machine learning algorithms to detect polyps [4, 5]. Efforts to solve the second problem have been more sporadic. Because we believe access to a public dataset will encourage research in the field, we make our data available. In this work, we present our approach to obtaining depth during colonoscopy as a step towards a full 3D model of the colon that can (a) provide real-time guidance guaranteeing the observation of the full colon and (b) help train colonoscopists to evaluate the percentage of the colon examined, which, in turn, can serve as a measure of quality during training.

## Related work

### A review of proposed methods for 3D reconstruction of the colon

Image-based monocular 3D reconstruction has drastically improved over the last decade with learning algorithms and improved computational power [6]. Nonetheless, 3D reconstruction in endoscopy and laparoscopy remains a difficult and open problem because endoluminal tissue is view dependent and reflective with limited texture which makes sequential image matching very challenging. Supervised approaches, on the other hand, remain infeasible as the colonoscope cannot easily integrate additional sensors, because it must be thin, flexible and has to encompass channels for water, air and instruments. This means that ground truth data that can train learning-based algorithms cannot be obtained using standard equipment and insufflation during colonoscopy deforms and invalidates anatomical data obtained from a computer tomography (CT) scan of the colon.

Approaches to 3D reconstruction of the colon and other parts of the gastrointestinal tract have traditionally not been data driven and have focused on modelling cues and characteristic geometry [7]. Both Structure-from-Motion (SfM) and Shape-from-Shading (SfS) as well as their combination have been considered alongside surface modelling [8]. More recently, learning methods have emerged using training data from a simulator that provides ground truth camera motion [9], or learning correspondences that are used to learn camera motion in an unsupervised manner [10]. The use of synthetic data generated from an anatomical CT scan or data from photorealistic phantoms has been explored to leverage data-driven approaches [11, 12]. Such approaches are trained on rendered images instead of real colonoscopy images and therefore require training of unsupervised transformer networks that translate between the two different domains. A fully unsupervised approach trained on sequential frames was shown to successfully predict relative depth information that can be used for polyp detection [4]. A self-supervised method, which embeds a depth prediction neural network in an SfM approach, trained on sequential images as well as sparse 3D points and relative camera poses obtained from SfM has also recently been proposed [13].

### An introduction to conditional generative adversarial models

Generative adversarial networks (GANs) [14] learn the probability distribution over a dataset. A standard GAN consists of two players: the generator, which learns to generate new, realistic looking instances, and the discriminator, which learns to distinguish the generator’s fake data from real data. Competing with one another, both players gradually improve, until the generator’s outputs are indistinguishable from the underlying real data.

**Fig. 1:**
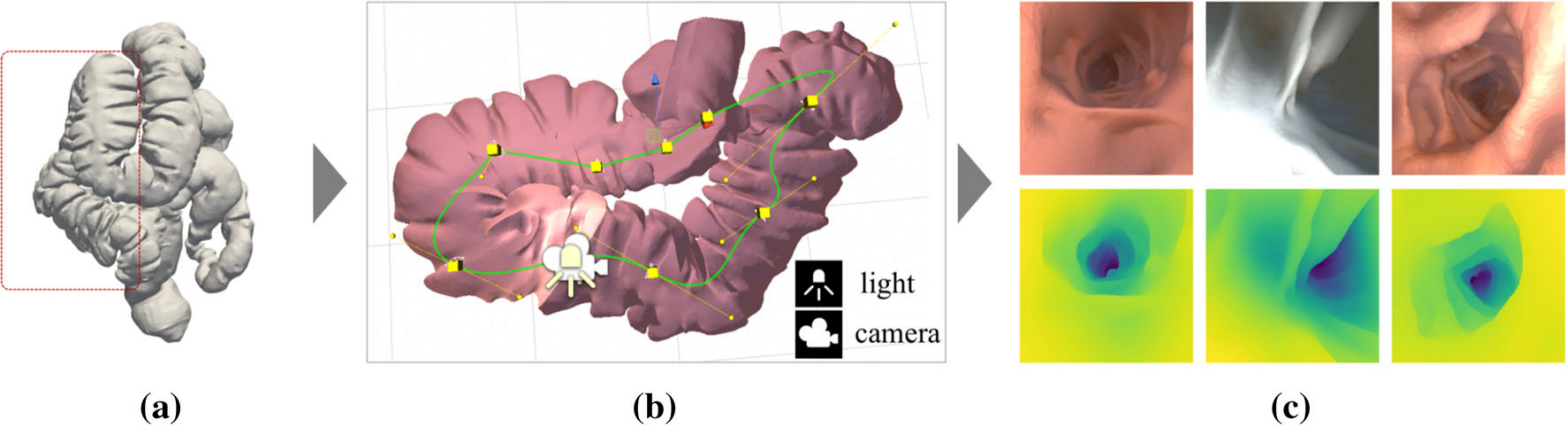
Synthetic data generation pipeline showing: **a** surface mesh of colon from computer tomography (CT); **b** red segment within the Unity environment with a virtual camera, camera path and light source; **c** examples of rendered RGB images with corresponding depth maps generated along camera path

Let $y = {\mathcal {G}}(z|\theta _g)$ describe the output of the generator function with random input $z\sim p_z$, where ${\mathcal {G}}$ is a convolutional neural network (CNN) with parameters $\theta _g$ and *z* is random noise. Further, let $p = {\mathcal {D}}(y|\theta _d)$ be the scalar output of the discriminator ${\mathcal {D}}$ with some input *y* and CNN parameters $\theta _d$, describing how probable it is that *y* is drawn from the real distribution. Then, the generator’s goal is to become so good at fooling the discriminator that the discriminator assigns high values $p = {\mathcal {D}}( {\mathcal {G}}(z|\theta _g)|\theta _d)$ to the outputs of the generator. Simultaneously, the discriminator is trained to assign small values to the outputs *y* of the generator, while assigning high values to real inputs $x\sim p_\mathrm{data}$. That is, generator and discriminator play a minimax game yielding at their equilibrium a generator

1 $$\begin{aligned} {\mathcal {G}}^*= & {} \arg \min _{\mathcal {G}}\max _{\mathcal {D}}{{\mathbb {E}}_{z\sim p_z(z)}[\log 1 - {\mathcal {D}}({\mathcal {G}}(z))]} \nonumber \\&+\, {{\mathbb {E}}_{x\sim p_\mathrm{data}(x)}[\log {\mathcal {D}}(x)]} \end{aligned}$$

2 $$\begin{aligned}=: & {} \arg \min _{\mathcal {G}}\max _{\mathcal {D}} {\mathcal {L}}_{GAN}({\mathcal {G}},{\mathcal {D}}) \end{aligned}$$

that ideally learns the distribution over the input data. For a proof of convergence, we refer to [14].

In the case of conditional GANs [15], the discriminator and the generator not only observe *y* or *z*, respectively, but also the condition *c*. Recently, different versions of cGANs have been proposed. Some condition on semantic labels [16], others, like pix2pix [17], learns to transform images between domains by conditioning on entire images. Given a set of correspondences (*c*, *x*) between real images and their depth maps, one can compare the output of ${\mathcal {G}}$ to its actual label by measuring the $L_1$-error. This error can be used to train ${\mathcal {G}}$ to generate outputs that not only look realistic, but are as close as possible to the real label. The loss functions are then

3 $$\begin{aligned} {\mathcal {L}}_\mathrm{cGAN}({\mathcal {G}},{\mathcal {D}}):= & {} {{\mathbb {E}}_{z\sim p_z(z),c\sim p_\mathrm{data}(c)}[\log 1 - {\mathcal {D}}(c,{\mathcal {G}}(c,z))]} \nonumber \\&+\, {{\mathbb {E}}_{c,x\sim p_\mathrm{data}(c,x)}[\log {\mathcal {D}}(c,x)]}, \end{aligned}$$

4 $$\begin{aligned} {\mathcal {L}}_{L_1}:= & {} \ {{\mathbb {E}}_{z\sim p_z(z), c,x\sim p_\mathrm{data}(c,x)}|| x - {\mathcal {G}}(c,z)||_1}, \end{aligned}$$

and ${\mathcal {G}}^* = \arg \min _{\mathcal {G}}\max _{\mathcal {D}} {\mathcal {L}}_\mathrm{cGAN}({\mathcal {G}},{\mathcal {D}}) + \lambda {\mathcal {L}}_{L_1}({\mathcal {G}})$, where $\lambda$ is a regularizing weight.

While this approach requires explicit input–output pairs for training, other models can be trained on unpaired examples [18].

### Contribution

We propose to train a cGAN to translate real colonoscopy images to depth maps. Although cGANs have been used for depth prediction in other environments [19], previous approaches required the availability of training data consisting of RGB–depth pairs. Because training data are difficult to obtain during endoscopy, we avoid the necessity of paired colonoscopy images and corresponding depth maps altogether. Instead, we propose to train a pix2pix network on paired simulated data and unlabelled real images. Our approach leverages the availability of real images improving the generalization capabilities of the network while avoiding explicit domain translation networks. These networks are error-prone, due to the lack of an application-specific loss function, and are trained independently of the depth prediction network precluding latter from back-propagating its gradients to the former. A cGAN, on the other hand, allows optimization of domain adaption and depth prediction simultaneously and is independent of camera, lighting and patient. Additionally, we publish the, to the best of our knowledge, first dataset of synthetic colonoscopy images with corresponding depth here: http://cmic.cs.ucl.ac.uk/ColonoscopyDepth.

## Methods

### Data generation

#### Synthetic dataset

We generate synthetic data based on a human CT colonography (CTC) scan (Fig. 1) from which we extract a surface mesh using manual segmentation and meshing. To render RGB endoscopic simulation images and corresponding depth maps, an environment developed using the game engine Unity is used. A virtual camera with two attached light sources, one on each side of the camera, can be scripted to follow a desired path through the virtual model. To vary textures and lighting conditions, we iteratively run render passes to generate nine data subsets. Each set represents a different configuration of three lighting conditions and one of three different materials. The materials vary in colour, reflectiveness and smoothness. The light sources vary in spot angle, range, colour and intensity. Randomly shifting and rotating the camera path with respect to the initial path, we obtained more than 16,000 images and depth maps with a maximum depth of 20 cm.

**Fig. 2:**
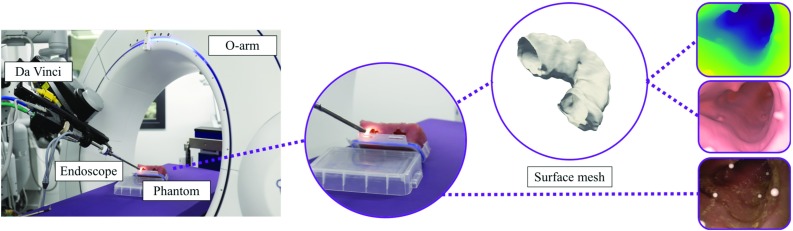
Setup for generating validation data showing from left to right: experimental setup; phantom; extracted 3D model; registered depth maps; CT renderings; and endoscopic images

#### Specularities

To achieve robustness towards reflections in real colonoscopy images, we augment our training data with random reflections. Although real specularities contain information about the surface normals, we observe that the network does not learn a relation to the shape. Keeping the depth maps unaltered, the networks learn to ignore specular reflections instead. With this approach, we eliminate the necessity of preprocessing images by removing reflections.

#### Generating ground truth data from a phantom

To test our algorithm on textured images captures with an endoscope, we collected data from a phantom. To make the phantom, a model of a colonic lumen was abstracted from a patient CT scan. Using this model, a press mould was 3D printed, which would allow for a negative mould to be made out of retractable plasticine. The negative mould was then placed in a box and filled with PVA-C.

Our setup for the ground truth generation is shown in Fig. 2. We fixed 3D markers at non-aligned positions inside the phantom. With the Medtronic O-arm Surgical Imaging System, we obtained a cone beam CT scan of the phantom. Using the da Vinci Surgical System, we positioned a stereo endoscope into the phantom and recorded image frames. After undistorting the left and right frames, we found the centres of the spherical markers by estimating their centroids in the image. We then triangulated the position of the markers to obtain 3D world coordinates for each marker. To get a 3D model, we generated a surface mesh from the CT scan using ITK-SNAP [20] and smoothed the result to reduce artifacts in the model reconstruction. The mesh was loaded into Unity, and we manually located the 3D positions of the markers in Unity coordinates. We estimated the world-to-Unity mapping using Procrustes analysis and applied it to the position of the left camera in the world reference frame. This gave us the position of the camera in Unity coordinates. Lastly, we replicated the intrinsic parameters of the left camera in Unity, resulting in a virtual world in which camera and surface mesh were aligned, and rendered depth maps from the endoscope’s point of view.

In theory, we should obtain an exact reproduction of the real-world scene, but errors are unavoidable due to inaccuracies in (1) the camera calibration; (2) the cone beam CT scan of the model; (3) the localization of the markers in the images and the CT model; (4) the projection matrix between real world and Unity environment based on four to seven correspondences. Such errors propagate and make it challenging to perform a perfect alignment of the real and synthetic scenarios.

Because the da Vinci endoscope is rigid, we can only image the phantom from a restricted set of angles. We display a representative view inside the phantom in Fig. 2. It can be observed that details in the phantom, like folds, are very smooth as opposed to the surface we extracted from the patient CTC. The images also have a narrower field of view than colonoscopy images from wide angle lenses. We therefore generated a second synthetic dataset consisting of about 5000 images using Unity in the same manner as our original training data replacing the human CT scan with the scan of the phantom.

**Fig. 3:**
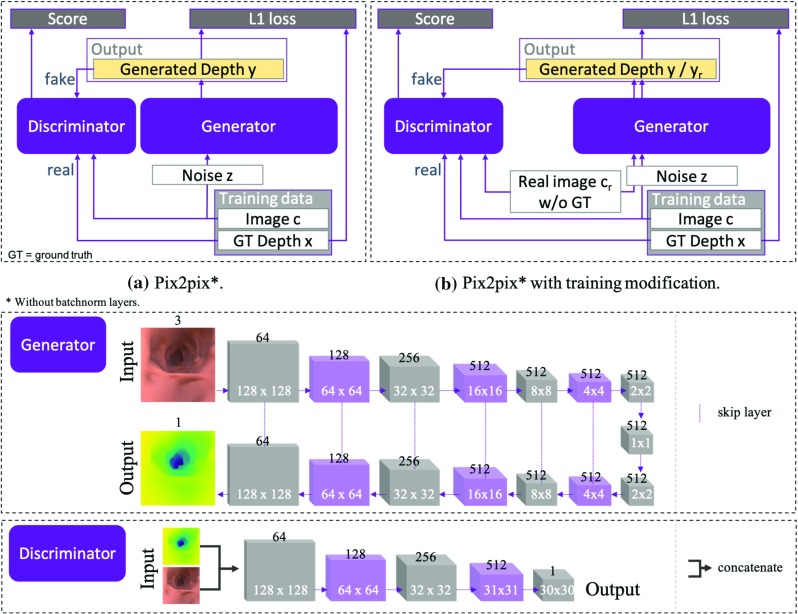
CNN architectures of standard and extended pix2pix model

### Model

GANs allow to learn the distribution over the shape of the colon, and while the texture of the synthetically generated data will differ from real data, the shape of a colon model generated from a CT mesh will be consistent. Therefore, learning a distribution over shapes observed during colonoscopy, the generative model can be trained to predict coherent depth maps, that is, even if it is exposed to unseen textures. Discriminative models, on the other hand, learn a deterministic mapping, which can fail once the input deviates strongly from the training data.

Let *c* equal an input RGB image, and *x* equal its corresponding depth map. Then, *y* is the predicted depth that the generator produces. A scheme of pix2pix is shown in Fig. 3a. The original version is solely trained on image–depth pairs (*x*, *c*), and instead of inputting a random vector *z* drawn from a normal distribution, dropout layers with dropout probability 0.5 are used during training and inference to simulate noise. The generator is composed of an encoder–decoder architecture with skip connections between each layer *i* and $L+1-i$ where *L* is the number of layers. Combining several levels of down-scaling enables the network to learn different degrees of details, with the first layer learning a general representation, and subsequent layers learning more local feature representations. The architecture can be found in [21]; however, we removed batch normalization layers because we noted those to cause inconsistent results and an exploding generator loss.

#### Extension

The cGAN learns to predict depth according to two criteria: due to the $L_1$ loss, the model learns to interpret cues from shading information—like a discriminative model would. Due to the cGAN loss, the model learns a prior over the expected shape. Both criteria can lead to over-fitting to synthetic data. Predicted depth maps from real images tend to be patchy, and shades that are thrown by folds or polyps are misinterpreted as high depth. This is due to the different texture of the synthetic training data. However, increasing the weight of the cGAN error to enforce a realistic shape has a downside. There are two differences to real shapes that have not yet been considered: firstly, synthetic images have a stair-step depth which is characteristic for CT data. Over-fitting a cGAN model to synthetic images, stair-step artifacts are visible even when predicting depth from real images. Secondly, a fixed pinhole camera without skew and offset was assumed to generate the synthetic dataset. Real colonoscopes have different intrinsics.

**Table 1:** Comparison of different models tested on synthetic data

Method	Mean $L_1$-error*	Mean relative $L_1$-error**	Mean RMSE*
Discriminative model	$0.234 \pm 0.047$	$8.4 \pm 2.1$	0.239
pix2pix	$0.232 \pm 0.046$	$8.2 \pm 2.0$	0.236
Extended pix2pix	$\mathbf 0.171 \pm 0.034$	$\mathbf 6.4 \pm 1.7$	$\mathbf 0.175$

*in cm; **in %

Bold values indicate the best performing method

**Fig. 4:**
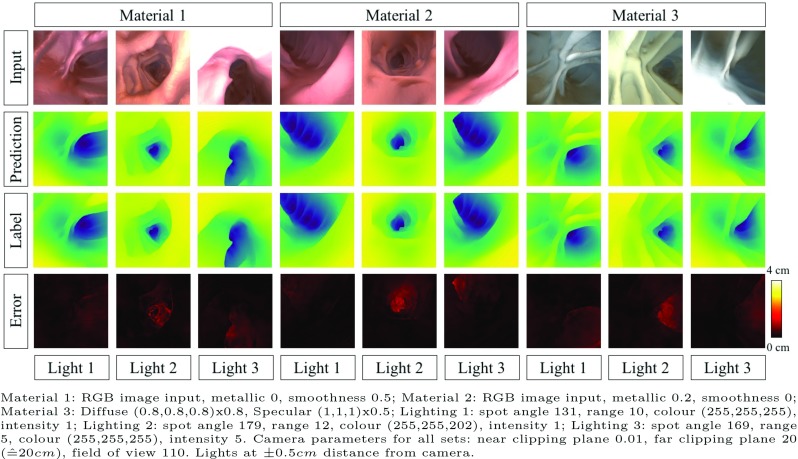
Results on synthetic data. Examples show different lighting on different materials

This results in a trade-off between constancy, that is, the ability to predict sensible shapes and to ignore misleading shading signals, and flexibility, for example the capability to predict depth from new domains or unseen shapes. To regularize for this trade-off, we suggest a training modification as shown in Fig. 3b. Additional to the synthetic training data, we input real images $c_\mathrm{r}$ to the model while excluding their prediction $y_\mathrm{r} = {\mathcal {G}}(c_\mathrm{r},z)$ from the $L_1$-error due to the missing labels. However, ${\mathcal {L}}_\mathrm{cGAN}$ still accounts for $y_\mathrm{r}$. Similar to a normal GAN, the generator will learn to predict a realistic looking depth map from real images. Without changing the weights of both losses, the model then learns to control for shades and unseen textures because the discriminator would identify deviating depth maps as fake. The model is implicitly adapting to a different domain. As a side effect, the shape-from-shading signal can now remain strong enough to prevent synthetic looking depths, without causing unrealistic depth maps.

#### Training

The model is trained in TensorFlow and takes about 12 h for training and less than 0.04 seconds for inference of one image on a NVIDIA Titan V GPU. We use Adam optimizer with a learning rate of $2\times 10^{-4}$ for both generator and discriminator and set the $L_1$ loss weight to $\lambda = 200$ as proposed in [17]. We train for 300 epochs using a batch size of 20. One element of each batch is a real example. We divide the dataset randomly into training, validation and test data using a 6:1:3 split, resulting in approximately 9600 training images.

## Experiments

### Synthetic data

We train and compare three models: (i) the original pix2pix model without batchnorm layers; (ii) pix2pix with our training modification which, for simplicity, we refer to as extended pix2pix, although we would like to emphasize that is not an extension to the model itself but only to the training; and (iii) a discriminative model that has the same architecture as the generator in the original pix2pix network. We measure the absolute $L_1$ distance $||\mathbf{x }-\mathbf{y }||_1$, the relative error $||\frac{\mathbf{x }-\mathbf{y }}{\mathbf{x }}||_1$ and the root-mean-squared error RMSE = $\sqrt{\frac{1}{N}\sum _n(x_n-y_n)^2}$ between ground truth depth *x* and predicted depth *y*. Results on all three models are shown in Table 1.

**Fig. 5:**
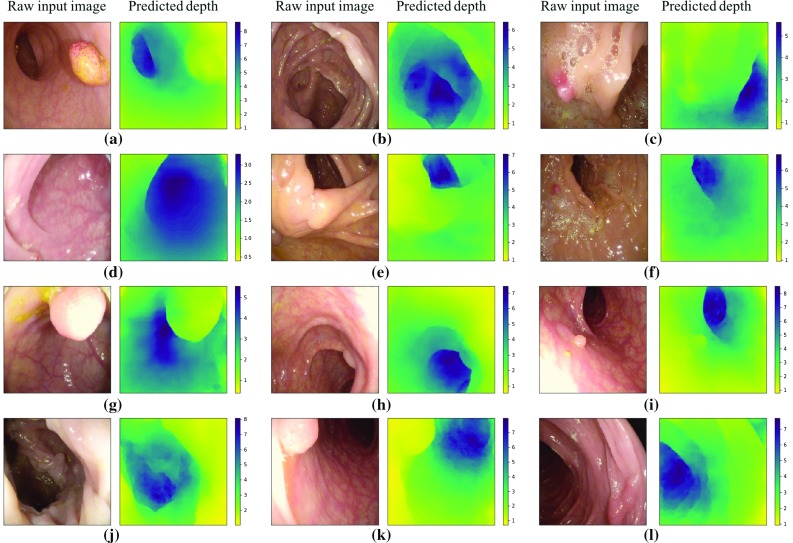
Results on real colonoscopy images showing accurate prediction of colonic lumen, polyps and folds despite specularities, blood vessels and air bubbles (all scales in cm)

We show that even on synthetic data the generative models lead to better results. The superiority of the extended pix2pix over the standard pix2pix on synthetic data is counter-intuitive but can be explained through the facts that the extension has a better ability to generalize and that hyper-parameters, which are crucial to the performance, were chosen on the extended model. Experimenting with input transformation, we find that logarithmically scaling the input depth and remapping it before the loss calculation has a beneficial result on the prediction. One reason could be that generators, in general, work less well on extreme values. Example predictions from each of the nine subsets of the testing set are shown in Fig. 4. One can observe that errors are especially high for regions with a larger distance from the scope. The average maximum pixel-wise $L_1$-error per image is 2.18 cm and is mainly found along folds with a step in depth.

**Fig. 6:**
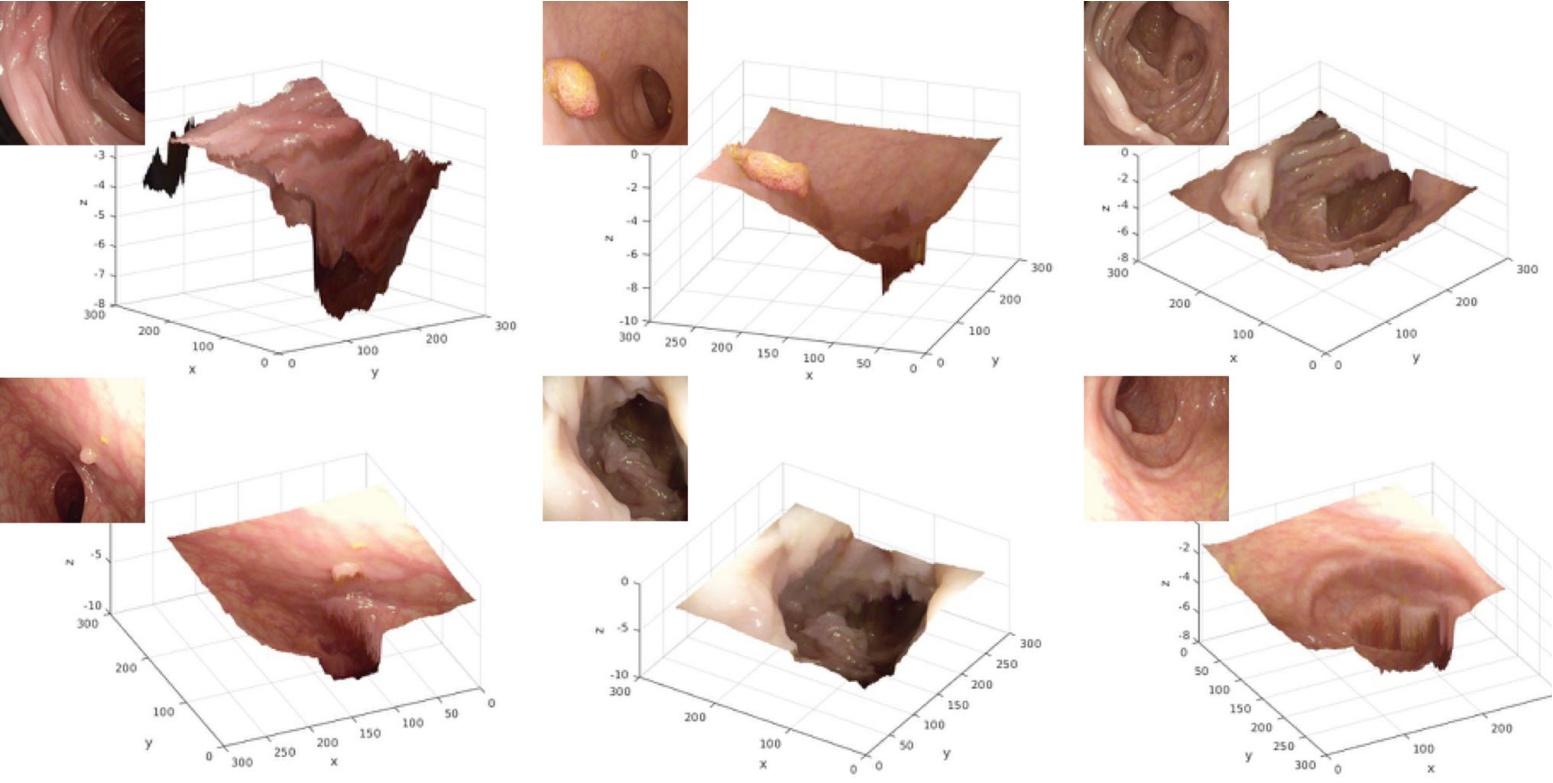
Colonoscopic images projected to 3D space (*x*-axis and *y*-axis in pixels, *z*-axis in cm): small details like folds and polyps have qualitatively correct relative depth

**Fig. 7:**
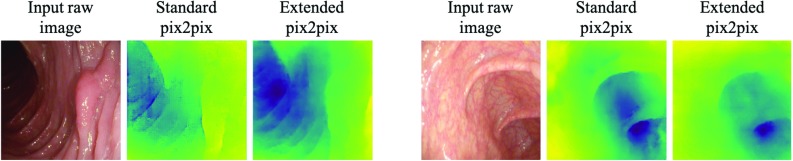
Comparison of standard pix2pix versus extended pix2pix: results using proposed training paradigm are smoother and more precise

**Fig. 8:**
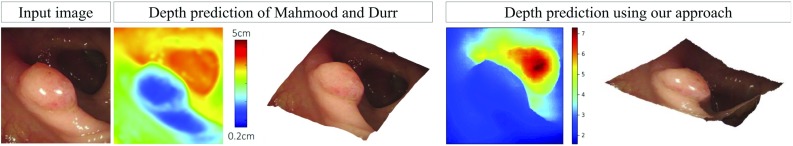
Comparison of proposed method and [12]. Three leftmost images adapted from [12] based on [23]. Training a cGAN emphasizes the lumen and overall tubular geometry

**Fig. 9:**
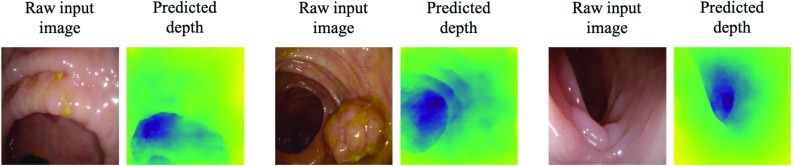
Examples for failed depth predictions on real colonoscopy scenes (5/44 scenes in which at least one frame fails according to qualitative measures) showing from left to right: wrongly predicted location of the lumen, missed polyp, misinterpreted geometry of the lumen

### Real data

We test the proposed training modification on real images without any preprocessing [22]. From Fig. 5, one can see the model detects minute details and, under qualitative inspection, predicts sensible depths. Critically, polyps and folds are correctly located. Even exceedingly small polyps, such as depicted in the centre of Fig. 5j, are correctly reconstructed. The images also reveal that reflections cause no inconsistencies nor do blood vessels or air bubbles, and hence, the model is largely invariant to texture. We further project some of the examples into 3D space and observe that the projected shapes are plausible (Fig. 6). From the images, one can see steps in depth along folds, the elevated nature of the polyps, and relative uniformity of the scale of the lumen across similar scenes.

**Table 2:** Comparison of different models tested on phantom data

Method	Mean $L_1$-error*	Mean relative $L_1$-error**	Mean RMSE*
Discriminative model	$1.969 \pm 0.040$	$33.9 \pm 0.3$	$2.207\pm 0.037$
pix2pix	$1.943\pm 0.112$	$33.0 \pm 1.8$	$2.202\pm 0.110$
Extended pix2pix	$\mathbf 1.417 \pm 0.128$	$\mathbf{24.7 } \pm 2.6$	$\mathbf 1.655 \pm 0.081$

*in cm; **in %

Bold values indicate the best performing method

**Fig. 10:**
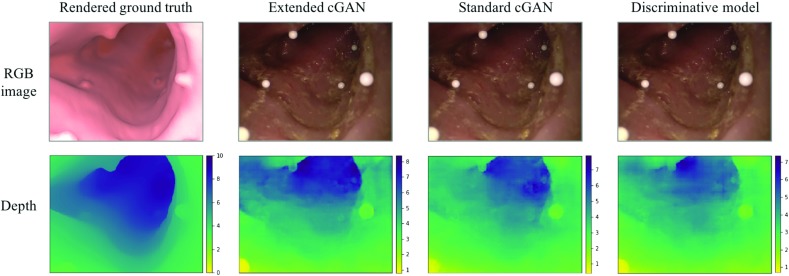
Validation of different models on phantom (all scales in cm). Although all models correctly locate the area of highest depth, the extended pix2pix network replicates the real shape and scale most accurately

We also compare the results using the new training paradigm with those from the standard version of pix2pix. Figure 7 shows that the depth maps generated by the standard version are uneven, less detailed and appear to be blemished. Further, we juxtapose our prediction to those of Mahmood and Durr [12] in Fig. 8. One can observe that our method appears to better reflect the overall tubular shape, the general depth, and the position and the magnitude of the lumen, although notably this is purely a qualitative statement and no full comparison is possible as we ran our method of images extracted from the pdf of their paper. Lastly, we show three out of five scenes (out of the 44 different testing scenes, each showing several frames) on which the extended pix2pix model fails in Fig. 9. The failures indicate that the model has learned to predict tubular shapes.

### Phantom

We trained the models on half of the original dataset and the additional dataset made from the phantom. The number of epochs was reduced to forty as the network starts to over-fit to the shape of the patient CTC. However, the synthetic dataset based on the CT of the phantom is too uniform to suffice as the sole training set. For the extended version of pix2pix, we used unpaired frames acquired from the da Vinci as real input images. Due to the homogeneity of the validation set, we pick three representative frames for validation. Results are shown in Table 2. Although the phantom is obstructed by markers, the network nevertheless recognizes the phantom’s shape (Fig. 10). Although the scale is inaccurate in the centre of the phantom, the overall scale is correctly identified. The $L_1$ loss between ground truth and prediction is 1.42 cm and also accounts for the markers obstructing the image and the reconstruction error discussed in “Data generation” section. The RMSE of the world-to-Unity transformation alone is on average 0.12 cm. For comparison, we train the original pix2pix and its discriminative version in the same manner as our proposed model and report the result on the same frame in Fig. 10. The $L_1$ distance between ground truth and prediction for the two models is 1.94 cm and 1.97 cm, respectively. This reflects a more than 37% greater error when compared with the extended pix2pix model.

## Conclusions

In this paper, we present our approach to predicting 3D depth from endoscopic images with specific application to colonoscopy. To compensate for the lack of labelled training data, we generate data from a simulation environment and propose a training modification to the well-known cGAN pix2pix that addresses the issue of a shift in domains between real and synthetic. Unlike previous approaches, our method can predict depth directly from both real and synthetic images, without the need of transformer networks and preprocessing. We show that our method preserves small details while producing smooth and sensible looking results on real colonoscopic images. While a quantitative evaluation on real data would be desirable, plausible but not metrically, accurate depth maps can have useful applications. Relative depth can help locate the lumen for navigation or be useful for SLAM approaches, which we will explore in our future work. Further clinical uses may involve measurement of size or distance relative to tools or polyps. Drawbacks of our approach are that the learned shape does not account for all scenes encountered during colonoscopy and that a lack of ground truth training data also means a lack of validation data. Our future work will focus on improving the registration process between real images and rendered images.
